# Dual Biopolymer Layer Using Nanoparticles with Active Substance Enclosed in Microcapsules: Innovative Solution for Slow Release of *Ginkgo biloba* L. Extract for Potential Therapies

**DOI:** 10.3390/ijms26073066

**Published:** 2025-03-27

**Authors:** Przemysław Sitarek, Monika Owczarek, Tomasz Kowalczyk, Wirginia Kukula-Koch, Magdalena Lasoń-Rydel, Lucyna Herczyńska

**Affiliations:** 1 Department of Medical Biology, Medical University of Lodz, Muszyńskiego 1, 90-151 Lodz, Poland; 2 Łukasiewicz Research Network—Lodz Institute of Technology, Skłodowskiej-Curie 19/27, 90-570 Lodz, Poland; monika.owczarek@lit.lukasiewicz.gov.pl (M.O.); magdalena.rydel@lit.lukasiewicz.gov.pl (M.L.-R.); 3 Department of Molecular Biotechnology and Genetics, Faculty of Biology and Environmental Protection, University of Lodz, Banacha 12/16, 90-237 Lodz, Poland; 4 Department of Pharmacognosy with Medicinal Plants Garden, Medical University of Lublin, Chodzki 1, 20-093 Lublin, Poland; virginia.kukula@gmail.com; 5 Textile Institute, Faculty of Material Technologies and Textile Design, Lodz University of Technology, Żeromskiego 116, 90-924 Lodz, Poland; lucyna.herczynska@p.lodz.pl

**Keywords:** microcapsules, nanotechnology, biopolymers, drug release, mathematical models, medical applications

## Abstract

The dynamic development of various branches of medicine and pharmacy, along with the emergence of new preventive and alternative therapies for various diseases, creates opportunities for new solutions utilizing carriers of active substances. Their therapeutic effect may occur through direct contact with skin lesions or indirectly, where medicinal substances penetrate the capillary network in the deeper layers of the skin and reach the bloodstream. The aim of the research was to obtain carriers with a matrix consisting of two renewable-source polymers (chitosan and ethylcellulose) and a core material derived from *Ginkgo biloba* green leaf extract (GBE). The obtained ethylcellulose microcapsules with encapsulated chitosan nanoparticles with extract {Et[Ch(GB)NP]} were characterized with respect to size, shape, surface morphology (SEM microscopy), and active substance release kinetics (UV-VIS and mathematical release models). The kinetics of active substance release were analyzed using UV-VIS spectroscopy and mathematical release models. The released active components were assessed microbiologically for activity against six bacterial strains and two fungal strains, as well as chromatographically using HPLC-ESI-QTOF-MS/MS fingerprinting. The microcapsules with a dual polymer layer exhibited a slow release of the core material, which demonstrated microbiological activity. The strongest antimicrobial effects were observed against *Klebsiella pneumoniae* and *Salmonella enteritidis*, with a minimum inhibitory concentration (MIC) of 410 µg/mL. The release of the core material from the double-layer polymer structures was more efficient in a physiological saline environment, with the best fit for the extract release kinetics following a zero-order model (regression coefficient R^2^ = 0.9939). The obtained microcapsules with a dual polymer layer show great potential for therapeutic applications in the medical industry. Their controlled release properties and antibacterial effectiveness make them a promising carrier for active substances in modern therapies.

## 1. Introduction

In recent years, there has been huge progress in nanotechnology, which has opened up new opportunities in various industrial sectors, including medical, pharmaceutical, veterinary and agricultural. One of the key areas of research is the development of effective drug delivery systems that can increase bioavailability and stability of the drug [1,2,3,4]. Traditional methods of delivering active compounds have several limitations, including rapid degradation, poor bioavailability, and difficult absorption. In addition, the hydrophobic or hydrophilic nature of some compounds can hinder their penetration through the skin or other biological barriers, reducing their therapeutic potential. These limitations fully justify the need to develop advanced delivery systems that protect bioactive compounds from degradation and increase their bioavailability [5,6,7]. In this field, nanoparticles have appeared to be effective carriers of active compounds.

Polymeric nanoparticles can be prepared from various types of polymers, which should be biocompatible with human cells and tissues that are non-toxic, non-antigenic, and biodegradable. Biodegradation plays a key role in the pharmacokinetic profile through prolonged release, subcellular size, and biocompatibility with human tissues [8,9]. Nanomaterials are widely used as drug carriers in cancer therapy, where their properties allow for precise delivery of drugs to cancer cells. Additionally, nanoparticles are used in diagnostics—for example, in biosensors—where they can increase the sensitivity and specificity of detection of disease markers [4,10,11]. Among various natural polymeric nanocomponents, chitosan-based nanoparticles are most often applied for topical application to the skin.

Chitosan is a natural biodegradable cationic polymer with antioxidant, anti-inflammatory, and antimicrobial properties. Ionic gelation is most frequently described and one of the most efficient methods of obtaining chitosan nanoparticles. Polycationic chitosan is dissolved in an aqueous solution of acid to which a negatively charged ionic cross-linking agent, usually pentasodium triphosphate (TPP), is added under intensive stirring [12,13,14,15]. In addition, the process can be carried out at room temperature, and the final size of the nanoparticles can be regulated by changing the chitosan/TPP ratio. Nanocarriers of natural origin are becoming more and more widely used due to their advantages, and the possibility of encapsulating biologically active compounds in them opens up more possibilities for their effective use [12,16,17]. An extremely important aspect is also the enhancement of the biological effect of, among others, plant extracts or pure compounds in combination with nanoparticles when developing new therapeutic strategies. This is particularly important in medicine, because many plant compounds have antioxidant, anti-inflammatory, or antibacterial properties, which can make this effect stronger. Such a strategy has already been used in the case of many plants with confirmed medicinal properties [18,19].

*Ginkgo biloba* L. (also known as maidenhair tree) is a relic and endemic species and the only one to survive the Cenozoic era. The leaf extract is a source of valuable metabolites, among which we can include flavonoids, terpene lactones, terpenoids, polyphenols, and others. GBE is used in people with excessive nervousness and also in cases of memory problems, dementia, and concentration disorders. In addition, it has antioxidant, anticancer, antimutagenic, and anti-asthmatic effects, and it accelerates wound healing process [20,21,22]. Our previous studies confirm that chitosan nanoparticles with *Ginkgo biloba* extract have a stronger cytotoxic effect on HeLa and PEA1 cancer lines than the extract alone. Additionally, no cytotoxic effect was demonstrated on normal cell lines in the tested concentration range [23].

Microencapsulation is currently one of the most dynamically developing technologies based on immobilization, i.e., coating or enclosing a small amount of an active substance by another material or a system of materials with different physicochemical properties. This process occurs by coating with a thin layer of a semipermeable polymer film, leading to formation of individual capsulesa composite product—or by locating the active substance in the polymer matrix as a result of occlusion and/or adsorption [24,25]. The most common methods for obtaining microcapsules and microspheres include emulsion methods, where emulsions are liquid dispersed systems in which one or more liquid phases are dispersed in the continuous phase, with these phases being insoluble in relation to each other. They have a very developed interphase surface, and they are thermodynamically unstable and easily destabilized and stratified. An emulsion is a type of dispersion system, where particles of the phase constituting the minority of the system (internal or dispersed phase) are dispersed in the external phase (dispersing), which constitutes the majority of the system as a result of mixing both phases [24,26,27].

Ethylcellulose, with a hydrophobic character, is one of polymers used to produce microcapsules. Cellulose derivative is quite widely used in the biomedical and pharmaceutical industries. Ethylcellulose skeleton is based on the repeating structure of the β-anhydride-glucose ring, which has three reactive functional groups (–OH groups). Due to its favourable biological properties, i.e., biocompatibility, it has been approved by the American Food and Drug Administration. This chemical substance is generally considered safe and exhibits potential to modulate and improve physiological properties of various bioactive compounds. In addition, it is used as a binder, a film-forming substance, in biopolymer forming coatings, and in implants and encapsulation [28,29,30].

**Figure 1:**
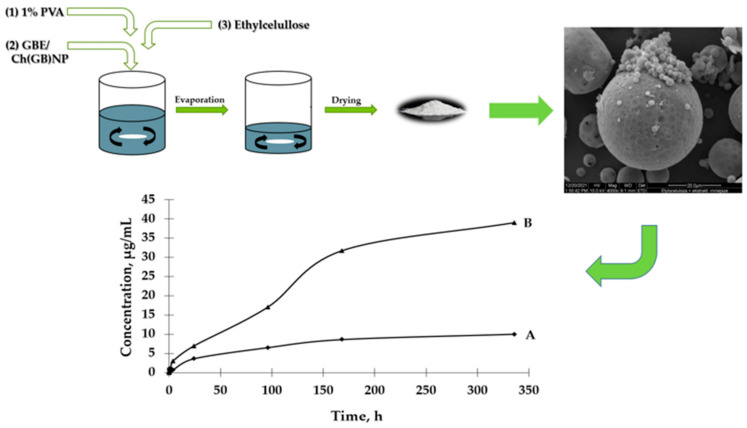
Schematic diagram of preparation of both type microcapsules, SEM imaging and GBE release.

The aim of this study is to prepare two types of functional microstructures: (1) ethylcellulose microcapsules with enclosed GBE [Et(GB)]; (2) microcapsules with a double polymer layer (ethylcellulose and chitosan with GBE (shown in Figure 1)) for a controlled and slow release of an encapsulated active ingredient (in this case, GBE) and antimicrobial evaluation of the obtained structures.

## 2. Results

### 2.1. Characterization of Ethylcellulose Microcapsules Et[Ch(GB)NP] and Et(GB)

**Figure 2:**
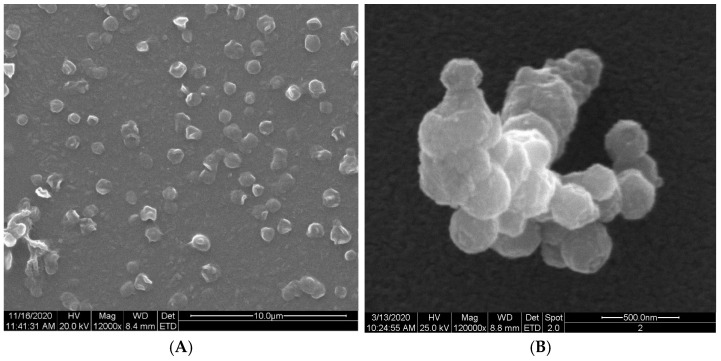
SEM images of Ch(GB)NP at 12,000× (**A**) and 120,000× (**B**) magnification.

**Figure 3:**
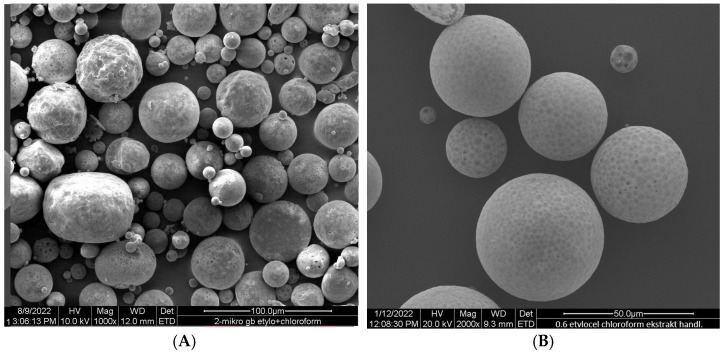

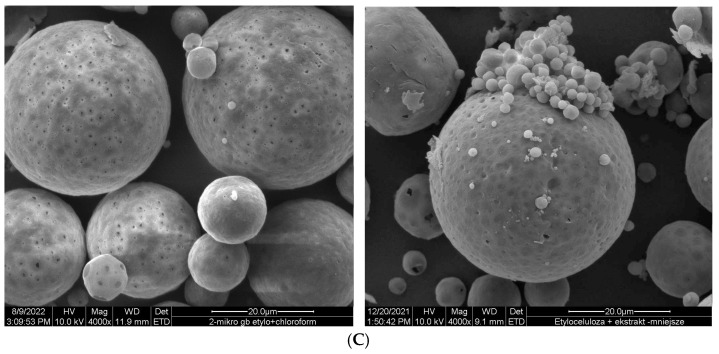
SEM images of Et(GB) at 1000× (**A**), 2000× (**B**) and 4000× (**C**) magnification.

**Figure 4:**
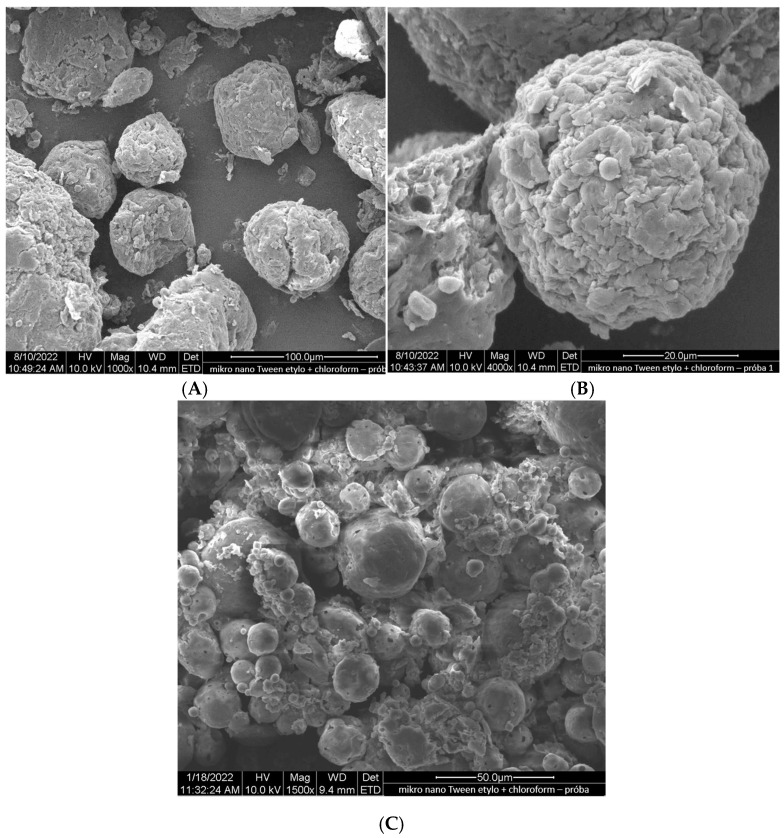
SEM images of Et[Ch(GB)NP] at 1000× (**A**), 4000× (**B**) and 1500× (**C**) magnification.

Ethylcellulose microcapsules with enclosed GBE—Et(GB) and Ch(GB)NP—Et[Ch(GB)NP] were obtained by the emulsion method with solvent evaporation. The average diameter of Ch(GB)NP (encapsulated in ethylcellulose microcapsules) in the tested sample was 454.2 nm (polydispersity index PDI = 0.549) [23]. The SEM image obtained and described in the previous publication by Owczarek et al. [23] is presented in Figure 2. The obtained capsules were imaged using the SEM technique. The presented Et(GB) was spherical with a quite smooth surface with numerous visible pores. The average diameter of the microcapsules from the measurement of the tested sample was 38.9 µm (Figure 3), while Et[Ch(GB)NP] are capsules of a very irregular shape with a rough surface, with an average diameter, from one measurement, of 40.6 µm (Figure 4).

### 2.2. GBE Release Rate from Et(GB) and Et[Ch(GB)NP]

#### 2.2.1. GBE Release Rate from Et(GB)

**Figure 5:**
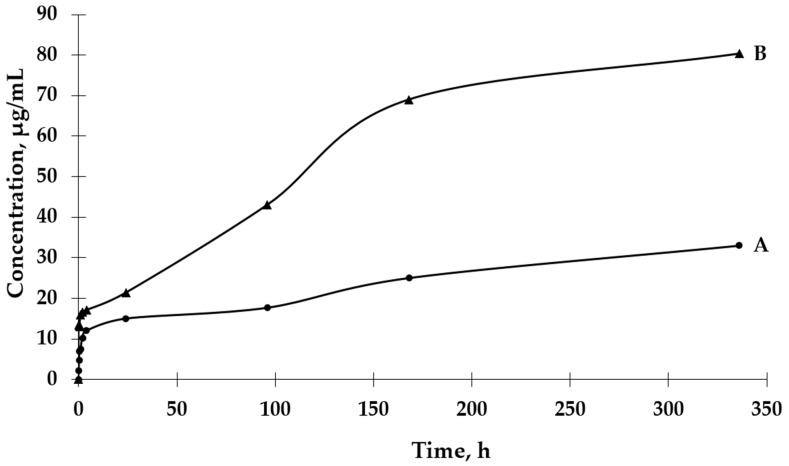
The release rates of GBE from Et(GB) in water (A) and physiological saline (B).

The UV-VIS spectrophotometric method was used to determine the GBE release rate from both types of microcapsules. The absorbance values for the peak characteristic for rutin were used to determine the concentrations of GBE released from the microcapsules (a standard curve of the dependence of the absorbance value on the GBE concentration was previously prepared). An analysis of the release curves of GBE from Et(GB) (Figure 5) reveals that there was an intensive release of the core material within 24 h. Then, in the following hours of the test, there were some episodes of a decrease in the release rate, but, on the whole, the release rate increased. Also, in this case, a more intensive release of the core material of the extract could be observed in the physiological saline medium than in water. Both GBE release curves from Et(GB) had an upward trend throughout the test.

#### 2.2.2. GBE Release Rate from Et[Ch(GB)NP]

**Figure 6:**
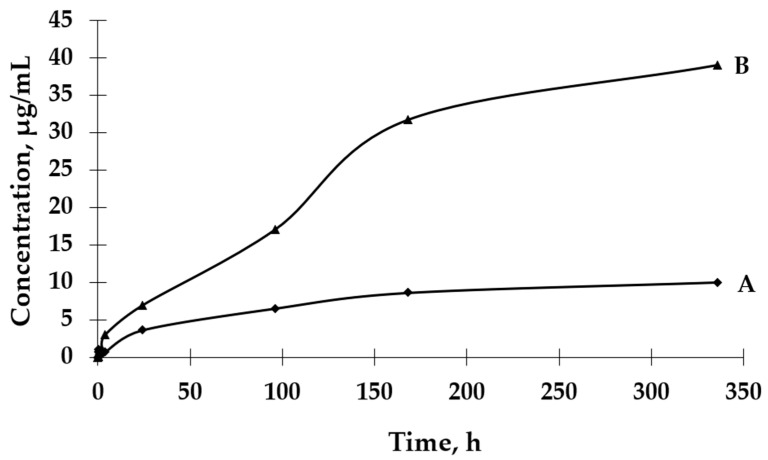
The release rates of GBE from Et[Ch(GB)NP] in water (A) and physiological saline (B).

Based on the results presented in Figure 6, it can be observed that the process of GBE release from Et[Ch(GB)NP] to the physiological saline environment had an increasing tendency during the entire test (a similar situation happened in the case of Et(GB)). Within 24 h, the release of the extract from Et[Ch(GB)NP] was quite intensive. Compared to the release of GBE from Et(GB), the amount of the released extract from Et[Ch(GB)NP] was relatively smaller. However, it was slightly less intensive compared to the release from Et(GB). The release of GBE from Et[Ch(GB)NP] to the aqueous environment increased very slowly until 168 h of the test, then stabilized and remained at a constant level, while in the physiological saline environment, it increased slowly.

### 2.3. Mathematical Models and Regression Coefficient Values of GBE Release from Nanoparticles Encapsulated in Cellulose Microcapsules

In order to better understand the drug’s release mechanism, the release profiles at pH 7.0 and 5.8 were analyzed by fitting the obtained release data to different kinetic models. To study the mechanism of GBE release, in vitro release profiles were correlated with various kinetic models, such as zero-order (cumulative amount of drug released vs. time), first-order (log cumulative percentage of drug remaining vs. time), Higuchi model (cumulative percentage of drug released vs. square root of time), and Korsmeyer–Peppas (log cumulative percentage of drug released vs. log time) release equations. The regression coefficient of GBE release from nanoparticles encapsulated in cellulose microcapsules are summarized in Table 1.

The results revealed good fitting profiles with the mathematical models. The best fit of the extract release kinetics in physiological saline solution was obtained using the zero-order model, while the release occurring in water best fit the Higuchi model.

The regression coefficient of GBE release from cellulose microcapsules are summarized in Table 2.

In the case of the release of the extract enclosed in cellulose microcapsules, the best fit was obtained for the zero- and first-order models when the process takes place in a physiological saline medium. For the release of *Ginkgo biloba* into water, the best fit of the kinetics was obtained using the Korsmeyer–Peppas model.

### 2.4. The HPLC-ESI-QTOF-MS/MS Fingerprinting of GBE

**Figure 7:**
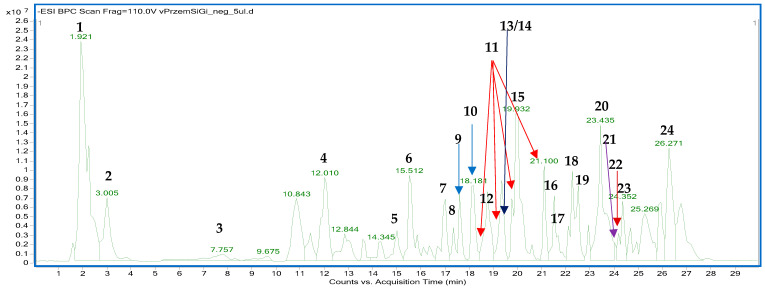
The fingerprint of GBE recorded in the negative ion mode with the indicated numbers of the tentatively identified metabolites as in the Table 3.

The fingerprinting aimed to prove the tested sample’s similarity to the previously described compositional data on *Ginkgo biloba* leaves. For the sake of this study, the fingerprint of the analyzed preparation was compared with the previously published data using a similar instrumentation. The performed HPLC-ESI-QTOF-MS/MS analysis showed a typical fingerprint of the *Ginkgo biloba* leaves that was in line with the publication of Zhong et al. [31]. As demonstrated herein, the commercially available ethanol–water tincture was rich in metabolites from different classes that were also present in the aforementioned publication. Among them, the most prominent peaks were due to the presence of a terpenic trilactone (bilobalide), phenolic acids, their glycosides, flavonoids, fatty acid esters, and characteristic components of *Ginkgo biloba* leaves, including ginkgolides, ginkgonic acids, and flavonoids like ginkgetin or amentoflavone. The list of tentatively identified compounds, along with their detailed spectral characteristics, is presented in Table 3 and Table S1 in the Supplementary File, whereas the fingerprint of the analyzed sample in the negative ion mode is shown in Figure 7.

### 2.5. Microbiological Tests (Determination of the Minimal Concentration Inhibiting the Growth of Microorganisms—Bacteria and Fungi)

**Figure 8:**
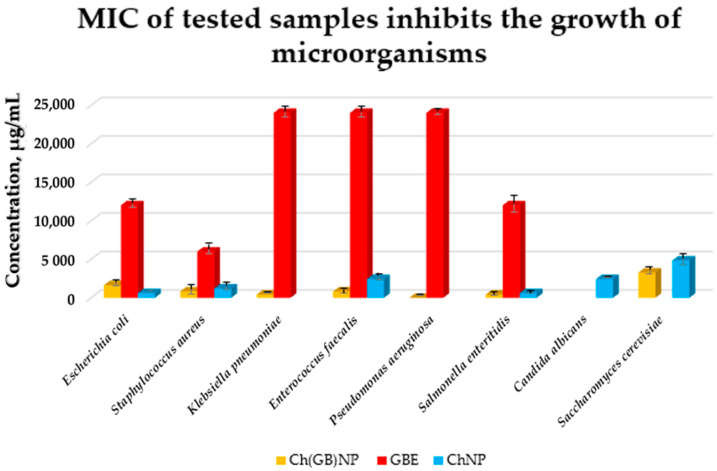
MIC of GBE, ChNP, and Ch(GB)NP inhibiting the growth of microorganisms (*Escherichia coli*, *Staphylococcus aureus*, *Klebsiella pneumoniae*, *Enterococcus faecalis*, *Pseudomonas aeruginosa*, *Salmonella enteritidis*, *Candida albicans*, and *Saccharomyces cerevisiae*).

The growth of microorganisms is identified by the appearance of turbidity or sediment in the liquid medium. Since the solutions tested in combination with the broth caused turbidity and sediment that made it impossible to read the result, the growth was assessed by sowing liquid cultures on an agar medium. The samples of pure extract (concentrated 10% *w*/*v* aqueous solution), empty chitosan nanoparticles (at a concentration of 17.2 mg/mL), and nanoparticles with closed extract (at a concentration of 24.2 mg/mL) were tested against eight strains of microorganisms (six bacterial and two fungal strains). The results of the micro-biological tests are presented in Figure 8.

Pure GBE showed antibacterial activity (the strongest against *Staphylococcus aureus* with MIC at 6000 µg/mL, intermediate against *Escherichia coli* and *Salmonella enteritidis* with MIC at 12,000 µg/mL, and the weakest against *Klebsiella pneumoniae*, *Enterococcus faecalis,* and *Pseudomonas aeruginosa* with MIC at 24,000 µg/mL). GBE showed no activity against fungi. ChNP showed activity against both bacteria and fungi (the strongest against *Escherichia coli* and *Salmonella enteritidis* with MIC at 610 µg/mL, less strong against *Staphylococcus aureus* with MIC at 1210 µg/mL, weaker against *Enterococcus faecalis* and *Candida albicans* with MIC at 2420 µg/mL, and the weakest against *Saccharomyces cerevisiae* with MIC at 4840 µg/mL). ChNP did not show microbiological activity against *Klebsiella pneumoniae* and *Pseudomonas aeruginosa*. In turn, Ch(GB)NP showed no activity, except against *Candida albicans*. However, they showed microbiological activity against the other microorganisms, i.e., starting from the strongest against *Klebsiella pneumoniae* and *Salmonella enteritidis* with MIC at a concentration of 410 µg/mL, strong against *Staphylococcus aureus* and *Enterococcus faecalis* with MIC at a concentration of 820 µg/mL, weaker against *Escherichia coli* with MIC at a concentration of 1630 µg/mL, and the weakest against *Saccharomyces cerevisiae* with MIC at a concentration of 3270 µg/mL. There is a visible effect of strengthening the antibacterial and antifungal activity as a result of using nanoparticles with the extract (except for *Escherichia coli*) compared to using empty chitosan nanoparticles as well as the extract alone.

## 3. Discussion

Nanotechnology and microencapsulation are rapidly growing areas of research in biology and biotechnology. Nanotechnology involves the use of nanometer-sized materials, allowing the precise manipulation of properties of substances and their targeted delivery to specific sites in the body. Microencapsulation involves the encapsulation of bioactive substances, such as plant extracts, in microcapsules to increase their stability, bioavailability, and protection against degradation under adverse environmental conditions. In biological research, microencapsulation allows the controlled release of active ingredients, which is particularly important in medicine, cosmetology, and the food industry. It can also reduce toxicity and improve the therapeutic efficacy of the studied extracts. The combination of these technologies opens up new possibilities for the development of innovative diagnostic and therapeutic solutions [32,33]. The aim of the present study was to prepare functional microstructures with a double polymer layer for the controlled and slow release of encapsulated GBE and to evaluate the antimicrobial performance of the obtained structures.

Microcapsules with an ethylcellulose shell have been prepared by an emulsion method using solvent evaporation. The size of the pores in the polymeric shell of the microcapsules directly affects the rate of release of the active ingredient contained in them and, consequently, on the possibility of controlling its dose according to potential applications. An analysis of release curves of the extract from Et(GB) and Et[Ch(GB)NP] and the release kinetics for both types of microcapsules shows an increasing trend (in saline environment) throughout the incubation period. In aqueous environments, the release stabilizes with time, especially for Et[Ch(GB)NP], while the release of GBE from Et(GB) increases slowly throughout the study period. This is probably due to the better solubility of GBE in saline (pH 5.8) than in water (pH 7.0). Prasertmanakit et al. showed that ethylcellulose microcapsules containing folic acid, prepared by emulsification, released up to 59% of their content within six hours of incubation at 37 °C in phosphate buffer and up to 70% of their content within 24 h of incubation under the same conditions [34]. In contrast, Yadav et al. showed that aceclofenac, an NSAID encapsulated in ethylcellulose microcapsules, released 70% of its total drug content within 6 h of incubation at 37 °C in phosphate buffer (pH 6.8) and in the range of 80–92% after 24 h of incubation [35]. Murtaza et al. [36] studied the release of diclofenac sodium from ethylcellulose microcapsules in aqueous environments. The study showed that 60% of the total diclofenac content in the microcapsules was released into the surrounding aqueous environment after 2 h and 20 min [36]. As the above studies [34,35,36] show, the rate of release of a substance from ethylcellulose microcapsules depends on the nature of the substance encapsulated in the microcapsule (hydrophilic or hydrophobic), which is then related to its affinity for the ethylcellulose shell and the medium into which it is released. The hydrophobic shell of the microcapsule allows the hydrophobic drug to be released more rapidly, whereas the hydrophilic carrier releases hydrophilic therapeutic substances more rapidly, as confirmed in previous studies [37]. The nature of the environment into which the therapeutic agent is released also influences the core material’s rate of migration. Hydrophilic compounds, such as aqueous plant extracts, have a natural tendency to diffuse very rapidly into the surrounding aqueous phase. Ethylcellulose, being a lipophilic polymer, slows down the diffusion process of hydrophilic compounds, but the surrounding hydrophilic test environment accelerates it [37]. Microencapsulation of chitosan nanoparticles with the extract significantly slowed the release of GBE into the environment. Studies conducted by Hasan et al. [37] confirmed that the use of microencapsulation of PCL (polycaprolactone) nanoparticles with encapsulated ibuprofen or triptorelin acetate using ethylcellulose reduced the release into the surrounding medium at 37 °C according to the following points:

- From 71.7% of ibuprofen content for nanoparticles in the 15th minute of incubation in the medium to 27.7% of ibuprofen content in ethylcellulose microcapsules with enclosed PCL nanoparticles (medium—phosphate buffer, pH 7.4);
- From 78.3% ibuprofen content for nanoparticles in 24 h of incubation in the medium to 57.3% ibuprofen content in ethylcellulose microcapsules with enclosed PCL nanoparticles (medium—phosphate buffer, pH 7.4);
- From 71.0% triptorelin acetate content for nanoparticles in the 15th minute of incubation in the medium to 5.4% triptorelin acetate content in ethylcellulose microcapsules with enclosed PCL nanoparticles (to 0.1 M NaCl medium); from 73.5% of triptorelin acetate content for nanoparticles in 24 h of incubation in the medium to 31.5% of triptorelin acetate content in ethylcellulose microcapsules with enclosed PCL nanoparticles (into 0.1 M NaCl medium) [37].

Fleczkó et al. [38] focused on comparing the release of vanillin from two types of ethylcellulose microcapsules: uncoated and coated with a cross-linked chitosan layer (release studies were conducted for 3 weeks at 50 °C). Both types of microcapsules showed prolonged release. However, the chitosan layer applied significantly prolonged the release of the core material. On day 6 of the experiment, the chitosan-coated microcapsules released approximately 20% of the total vanillin content, while the uncoated microcapsules achieved the same percentage release after only 1 day. After 2 weeks of testing, the uncoated microcapsules released 60% of the total vanillin content, while the coated microcapsules released just under 30% of the core material content. After 3 weeks, the uncoated microcapsules released 80% of the vanillin content, while the coated microcapsules released 30% of the total vanillin content [38]. The study confirmed that the ethylcellulose carrier for encapsulating chitosan nanoparticles with the extract, as well as GBE, plays a role in slowing the release of the extract into the aqueous environment and physiological saline solution. The release of GBE through a single ethylcellulose barrier (in the case of Et(GB)) and through two polymeric barriers: cross-linked chitosan and ethylcellulose {in the case of Et[Ch(GB)NP]} tends to increase (in saline) or stabilize over time (in aqueous medium), so that the polymers chosen as carriers of the active substance meet the expectations of a carrier for controlled and slow release over time.

Based on the kinetic modeling results presented in Table 1 and Table 2, the release of GBE from both types of microcapsules [with GBE and Ch(GB)NP] into physio-logical saline medium—pH 5.8, is best described by the zero-order and first-order release models. The best fit of the extract release kinetics in physiological saline solution was obtained for the zero-order model [regression coefficient is R^2^ = 0.9939 for microcapsules with extract and R^2^ = 0.9945 for microcapsules with Ch(GB)NP, respectively]. A good fit to the zero-order release model indicates release at a constant, sustained rate, thus maintaining the concentration of the active substance for a longer period of time [39,40,41,42]. A very good fit of the release kinetics of the extract from both types of microcapsules to physiological saline was obtained for the first-order release model (regression coefficient R^2^ = 0.9874 and R^2^ = 0.9913, respectively). The first-order release mathematical model is used to describe the dissolution process of the released active substance into the surrounding medium [39,40]. In the case of GBE release from ethylcellulose microcapsules into an aqueous medium, the best-fitting mathematical model is the Higuchi model (regression coefficient R^2^ = 0.9738); for microcapsules with Ch(GB)NP, the Korsmeyer–Peppas model (R^2^ = 0.9048) is used.

For the Higuchi release model, the main release mechanism is diffusion, which is important in the entire release process of the encapsulated active substance; dissolution significantly contributes to the initial phase of the process. After dissolution of the outer, more soluble coating of particles, the main process will be the internal diffusion of ions from the mass to the surrounding aqueous medium [43,44]. The Korsmeyer–Peppas kinetic release model is based on the principle of release of the active substance from the polymer matrix as a result of swelling [40]. Owczarek et al. proved that the best fit of the release kinetics of GBE enclosed in chitosan nanoparticles to physiological saline solution was obtained for the first-order model (regression coefficient R^2^ = 0.9104), and for the release to the aqueous medium, the Higuchi model (R^2^ = 0.9204) was used [23]. On the other hand, Jonassen et al. [45] showed in their studies that chitosan has a rigid and stretched conformation in water due to electrostatic repulsion between positively charged amino groups in chitosan chains. The charges are reduced when, for example, a saline solution is used as a medium instead of water. The screening effect in physiological saline environment reduces the electrostatic repulsion between positively charged groups in chitosan chains; therefore, these chains are more flexible in a physiological saline medium [45]. The release of GBE occurring through a single chitosan or ethylcellulose barrier (in the case of Ch(GB)NP or Et(GB)) and through two polymer barriers (cross-linked chitosan and ethylcellulose {in the case of Et[Ch(GB)NP]} tends to increase (in physiological saline) or stabilize over time (in an aqueous environment). Therefore, the selected polymers as carriers of the active substance meet the expectations of a carrier for a controlled and slow release over time.

Phytochemical analysis and identification by HPLC-ESI-QTOF-MS/MS fingerprint-ting of a commercial *G. biloba* leaf extract confirmed the presence, according to the available literature, of various classes of compounds among which are organic acids, carbonyls, fatty acids, furanones, phenols, vitamins, and alkylphenols. Many of these compounds exhibit a range of biological properties, such as anticancer, neuroprotective, antimicrobial, antioxidant, and anti-inflammatory [31,46,47,48]. Sanchez-Hernandez et al. demonstrated that active compounds contained in GBE, such as dihydro-4-hydroxy-2(3H)-furanone, 2,4-dimethyl-3-hexanol, catechol, 3-O-methyl-D-fructose, 4,6-di-O-methyl-α-D-galactose, 2-O-methyl-α-D-xylfuranoside, and 3-methyl-mannoside, exhibit antimicrobial activity [49]. Hua et al., on the other hand, showed that ginkgolic acid has significant antimicrobial activity against Gram-positive (G+) bacteria [50]. These compounds were also present in the extract we tested, which may confirm these properties. Yet, in combination with nanotechnology, these are the first reports on the subject. Minimum inhibitory concentrations (MIC) for GBE, pure chitosan nanoparticles, and Ch(GB)NP were determined for six bacterial and two fungal strains in liquid culture medium followed by agar plates. A lower MIC value indicates that a lower dose of the therapeutic agent is required to inhibit the growth of the microorganism; therefore, drugs with lower MIC results are more effective antimicrobial agents. By identifying appropriate agents and their corresponding effective concentrations, MIC assessments help prevent the development of drug resistance in different strains of microorganisms [51]. MIC tests were performed using a commercial extract concentrated to 10% *w*/*v* [23]. The used GBE showed antimicrobial activity in the tested concentration range. The lowest concentration of GBE was active against *S. aureus*; two times the concentration of the extract was active against *E. coli* and *S. enteritidis*, and four times the concentration inhibited the growth of *K. pneumoniae*, *E. faecalis,* and *P. aeruginosa*. The above concentrations were also found to be the minimum bactericidal concentrations (MBC), as confirmed by the lack of growth on agar media inoculated from broth cultures. In the tested concentration range, GBE showed no activity against fungal strains. Ibrahim et al. confirmed the antibacterial and antifungal activity of the standardized GBE (EGb 761) in their study [52]. They also showed that the MIC for *S. aureus*, *E. coli*, *K. pneumoniae*, *S. cerevisiae,* and *C. albicans* was 15 mg/mL, and the MBC for all strains (except *K. pneumoniae*) was 30 mg/mL (for *K. pneumoniae* the MBC was 60 mg/mL) [52]. Other researchers using *G. biloba* leaf extract have shown that a concentration of 50 mg/mL is the MIC and inhibits the growth of the following strains *E. aerogenes* ATCC 13048, *S. epidermidis* DSMZ 20044, *S. aureus* ATCC 25923, *E. coli* ATCC 25922, and *B. subtilis*, while a concentration of 100 mg/mL has a bactericidal effect on all these bacteria [53]. The mechanism of action of pure nanoparticles and Ch(GB)NP against G– and G+ bacteria is slightly different due to differences in the cell wall and cell membrane structure of the two groups of bacteria. The cell membrane of G– bacteria contains lipopolysaccharide (LPS) in its structure, which has numerous anionic groups, such as phosphate or pyrophosphate groups, which have an affinity for cationic chitosan. In G+ bacteria, the dominant outer cell wall is composed of murein (peptidoglycan) and teichoic acid residues. This clearly explains why the loss of intracellular content after PDO treatment is greater in G– than in G+ bacteria [54]. A thinner cell wall of G– bacteria makes them more susceptible to nanoparticle treatment, while the peptidoglycan layer in the G+ cell wall prevents the internal binding of chitosan nanoparticles [55,56]. In addition to the type of bacteria (G+ or G–), the antimicrobial activity of chitosan nanoparticles also depends on other factors, such as bacterial growth phase, zeta potential, concentration, pH, molecular weight, and degree of deacetylation [54]. Ch(GB)NP in the tested concentration range showed no activity, except against *C. albicans*. In contrast, chitosan nanoparticles with GBE showed microbial activity against the remaining microorganisms, i.e., starting with the strongest activity against *K. pneumoniae* and *S. enteritidis*. A two-times higher concentration was necessary to inhibit *S. aureus* and *E. faecalis*, a four-times higher concentration was used against *E. coli,* and an eight-times higher against *S. cerevisiae*. With regard to the minimum inhibitory concentrations for GBE and pure nanoparticles, the effect of enhancing the antimicrobial and antifungal activity, being a result of the extracted nanoparticles (except for *E. coli*), is evident. Studies on the microbiological synergistic effect of chitosan nanoparticles and GBE against bacteria and fungi constitute innovative research, as there are no literature references confirming this effect. The researchers focused on testing other agents (including plant extracts or drugs) in combination with the effect of chitosan nanoparticles. This solution was shown to enhance the effect of antibiotics (ciprofloxacin and gentamicin) by increasing the zone of growth inhibition around textile discs with different concentrations of antibiotics and chitosan nanoparticles with encapsulated antibiotics. The zone of growth inhibition of *S. aureus* around chitosan nanoparticles with ciprofloxacin discs increased by 94.7% compared to the zone of growth inhibition around ciprofloxacin discs at 50 mg/mL antibiotic concentration, while the zone of growth inhibition of *E. coli* around nanoparticles with ciprofloxacin discs increased by 94.2% compared to the zone around discs with the antibiotic at the same concentration. In the case of gentamicin, the zone of inhibition for both bacteria around the nanoparticle–gentamicin discs increased 12-fold for *E. coli* and 15-fold for *S. aureus* compared to the zone of inhibition around the discs with gentamicin (also at 50 mg/mL antibiotic concentration) [57]. Sotelo-Boyás et al. showed that the components in the form of chitosan nanoparticles and lime essential oil showed a synergistic effect in antimicrobial activity against the foodborne pathogens tested, i.e., L. *monocytogenes*, *S. dysenteriae*, *S. aureus,* and *E. coli* [58]. Another study demonstrated the synergistic effect of nanoparticles with curcumin against *S. aureus* and *P. aeruginosa* by conducting in vivo studies on mice. This study showed that chitosan nanoparticles loaded with curcumin significantly inhibited the progression of *S. aureus* and *P. aeruginosa* infection on mouse skin, while empty nanoparticles showed no such inhibition of infection [59]. The antimicrobial activity of nanoparticles with curcumin has been shown to be significantly superior to curcumin alone. Chitosan nanoparticles in drug delivery systems have reduced disadvantages of conventional delivery systems, probably due to a gradual release of curcumin by chitosan nanoparticles in infected areas. Therefore, chitosan nanoparticles in combination with curcumin produced a synergistic effect in antimicrobial activity that was more potent than either component alone [59].

## 4. Materials and Methods

### 4.1. Materials

The following materials were used in the study: chitosan (Primex, Chitoclear fg 95, average molecular weight (MW) ~ 234.55 kDa, DD = 86.8%, viscosity 99.17 cP measured for 1% aqueous solution at 20 °C, Siglufjordur, Iceland); acetic acid (Avantor, 80%, analytical grade, Gliwice, Poland); pentasodium triphosphate—TPP (Sigma–Aldrich–Merck, analytical grade, ≥98.0%, Cat. No.: 72061, Taufkirchen, Germany); commercial medical device Tinctura Ginkgo bilobae–water–ethanol extract of green *Ginkgo biloba* leaves (concentrated to the above 10% solution, Phytopharm, Nowe Miasto nad Wartą, Poland); Polysorbate 80—Tween 80 (Lach:ner, s.r.o., Neratovice, the Czech Republic); sodium chloride (Chempur, pure, Piekary Slaskie, Poland); polyvinyl alcohol—PVA, (POCh, average MW ~ 20,000 Da, Gliwice, Poland); ethylcellulose (Aldrich Chemistry, viscosity 4 cP measured in 5% toluene/ethanol 80:20 solution, ethoxyl, content 48%, St. Louis, MO, USA); chloroform (Chempur, pure, Piekary Slaskie, Poland); *Escherichia coli* ATCC 11229 (Biomaxima S.A., Lublin, Poland); *Staphylococcus aureus* ATCC 6538 (Biomaxima S.A., Lublin, Poland); *Klebsiella pneumoniae* ATCC 4352 (Biomaxima S.A., Lublin, Poland); *Enterococcus faecalis* ATCC 33186 (Biomaxima S.A., Lublin, Poland); *Salmonella enteritidis* ATCC 13076 (Biomaxima S.A., Lublin, Poland); *Pseudomonas aeruginosa* ATCC 9027 (Biomaxima S.A., Lublin, Poland); *Candida albicans* ATCC 10259 (Biomaxima S.A., Lublin, Poland); *Saccharomyces cerevisiae* (Lallemand Polska, Sp. z.o.o., Płochocin, Poland); Müller Hilton II Broth—MHB medium (BTL Sp. z.o.o., Łódź, Poland); Sabouraud medium with 4% glucose (BTL Sp. z.o.o., Łódź, Poland); PCA agar medium (BTL Sp. z.o.o., Łódź, Poland); bacteriological agar (BTL Sp. z.o.o., Łódź, Poland); and distilled water (Łukasiewicz–ŁIT, Łódź, Poland).

### 4.2. Preparation of Chitosan Nanoparticles with GBE and Their Characteristics

Chitosan nanoparticles with enclosed GBE [Ch(GB)NP] were obtained using the ionic gelation technique with the use of pentasodium triphosphate (TPP) as a cross-linking agent. The applied materials and methods of obtaining Ch(GB)NP and its characteristics were described in detail by Owczarek et al. [23].

### 4.3. The Fingerprinting Studies of GBE by HPLC-ESI-QTOF-MS/MS Approach

The tincture was filtered through nylon filters with an internal pore diameter of 0.1 µm and directly injected on a freshly calibrated chromatographic platform, namely HPLC-ESI-QTOF-MS/MS, produced by Agilent Technologies (Santa Clara, CA, USA). The instrument was composed of an HPLC chromatograph (1200 Series by Agilent Technologies, Santa Clara, CA, USA) with a degasser, an autosampler, a DAD detector, an isocratic pump for internal calibrant, a column thermostat, and a QTOF mass spectrometer (G6530B) with a dual AJS ESI ionization source. The separation of the tincture’s metabolites was achieved on an RP-18 Zorbax Eclipse Plus column (3.5 µm pore size, 150 mm × 2.1 mm) in a gradient method. Solvent A contained 0.1% aqueous formic acid, whereas solvent B was composed of 0.1% formic acid in acetonitrile. The following program was applied: 0 min—1% B, 10 min—20% B, 15 min—40% B, 17–22 min—95% B, 22.1–30 min—1% B. The injection volume was 5 µL, whereas the flow rate of the mobile phase was set as 0.2 mL/min from the beginning of the analysis until 22.1 min. It was then gradually increased up to 0.250 mL/min until 25 min and was kept until the end of the analysis at 30 min. The settings of the mass spectrometer included a 100–1200 m/z range, a gas temperature of 250 °C, a sheath gas temperature of 300 °C, a gas flow of 12 L/min, a nebuliser pressure of 35 psi, a fragmentor voltage of 110 V, a capillary voltage of 3000 V, a nozzle voltage of 1000 V, a skimmer voltage of 65 V, and collision energies of 10 and 20 V. A data-dependant experiment was run. Two m/z features with the highest intensity were fragmented in each scan at two of the aforementioned collision energies. After collecting two spectra, the fragmented molecular features were excluded for the following 0.3 min from fragmentation to give way to less intensive signals. Both the data acquisition and analysis were performed with the help of the Mass Hunter Workstation software (version B.12.00, Agilent Technologies). Sample fingerprinting, directed to a tentative assignment of the recorded ions, was performed while analyzing the high-resolution mass measurements, using MS/MS fragmentation pattern, with the help of the published scientific literature on the same plant, and using a similar methodology.

### 4.4. Determination of the Minimal Inhibitory Concentration of Microorganisms—Bacteria and Fungi

The MIC (minimal inhibitory concentration) method was used for the tests, which is a macrodilution method based on the method developed by Kowalska-Krochmal et al. [60]. The dilution technique in test tubes involves exposing microorganisms in a liquid medium to decreasing concentrations of antimicrobial agents obtained by serial two-fold dilution. The mixture, consisting of microorganisms, nutrient, and antimicrobial agent, was incubated at 35 °C for 16–20 h. The lowest concentration of antimicrobial agents, i.e., the concentration at which no visible growth of microorganisms occurred, was defined as the minimum inhibitory concentration (MIC). The following bacterial strains were used for microbiological tests: *Escherichia coli* ATCC 11229, *Staphylococcus aureus* ATCC 6538, *Klebsiella pneumoniae* ATCC 4352, *Enterococcus faecalis* ATCC 33186, *Salmonella enteritidis* ATCC 13076, *Pseudomonas aeruginosa* ATCC 9027, *Candida albicans* ATCC 10259, and *Saccharomyces cerevisiae* (by Lallemand Polska, Sp. z o. o.). In total, 3–5 colonies were taken from a 24-h bacterial reduction culture and inoculated with 5 mL of MHB medium (Müller Hilton II Broth). The inoculated media were incubated for 24 h at 35 ± 1 °C. Then, the culture was diluted to obtain a bacterial suspension with a concentration of 0.5 on the McFarland scale. The inoculum was obtained by diluting 0.5 McFarland suspension in MHB broth at a ratio of 1:100.

The yeast suspension inoculum was prepared by suspending, in a liquid medium (Sabouraud with 4% *w*/*v* glucose), colonies taken from the agar culture to obtain a concentration of 10^6^ cfu/mL (incubation for 24 h at 25 ± 1 °C).

**Figure 9:**
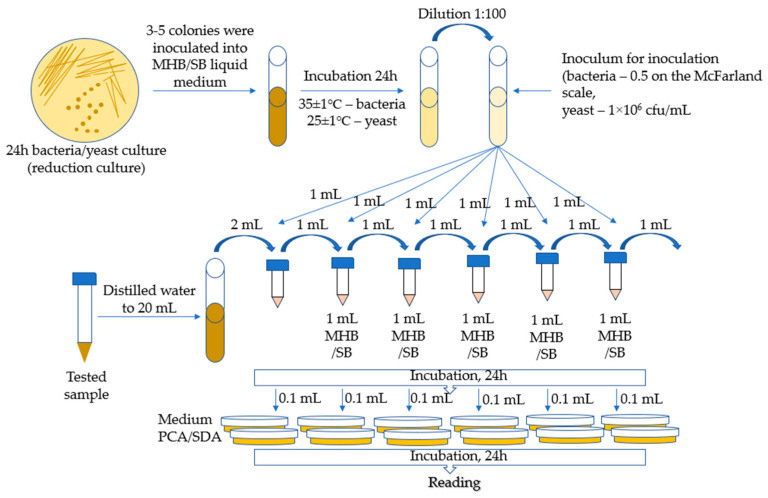
Schematic diagram of the MIC (for bacteria and yeast).

Samples of 5 mL each of Ch(GB)NP (initial concentration 24.2 mg/mL), ChNP (initial concentration 17.2 mg/mL), and GBE (extract with a concentration of 10% *w*/*v*) were supplemented with distilled water to a volume of 20 mL. Then, a series of two-fold dilutions were performed. The prepared series of solution dilutions were poured into test tubes in amounts of 1 mL, after which 1 mL of microbial inoculum was added to each tube. The broth inoculated with microorganisms with 1 mL of distilled water was left as a control. Incubation was carried out for 24 h at 25 ± 1 °C for *S. cerevisiae* and at 35 ± 1 °C for the remaining microorganisms. MIC tests were carried out in the following concentration ranges for the individual samples: GBE (750–24,000 µg/mL), ChNP (150–4840 µg/mL), and Ch(GB)NP (100–3270 µg/mL). The growth of microorganisms is identified by the appearance of turbidity or sediment in the broth. Since the solutions tested in combination with the broth caused turbidity and sediment, making it impossible to read the result, the growth was assessed by sowing liquid cultures on an agar medium. Liquid cultures in the amount of 0.1 mL were poured onto Petri dishes and covered with PCA agar medium for bacteria and SDA for yeast. After 24 h of incubation (under the same conditions as for the incubation of liquid media), the growth of microorganisms on the plates was assessed by comparison with the growth on the control. The MIC was assumed to be the lowest concentration of the active substance at which the growth of microorganisms was significantly lower than that of the control. The schematic diagram for determining the MIC (minimum inhibitory concentration for the growth of bacteria and yeast) is presented in Figure 9.

### 4.5. Preparation of Ethylcellulose Microcapsules {Et[Ch(GB)NP] and Et(GB)}

**Figure 10:**
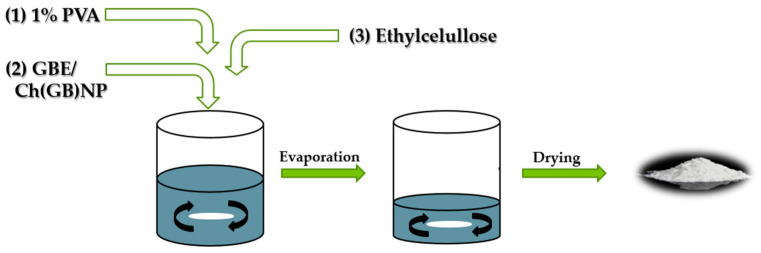
Schematic diagram of the process for obtaining Et[Ch(GB)NP] and Et(GB).

Microcapsules with ethylcellulose coating were obtained by the solvent diffusion method from emulsion. The O/W emulsion was prepared by homogenizing the organic phase (0.6 g of ethylcellulose was dissolved in 10 mL of chloroform) with the aqueous phase (40 mL) containing 5 mL of chitosan nanoparticles with GBE {to obtain ethylcellulose microcapsules containing chitosan nanoparticles—Et[Ch(GB)NP]} or 5 mL of GBE [to obtain ethylcellulose microcapsules containing extract—Et(GB)] and a surfactant, 1% poly(vinyl alcohol) solution. Microparticles were formed after the complete removal of the solvent from the droplets by evaporation under stirring (1400 rpm). The produced microcapsules were separated from the solution, washed, and dried (at room temperature). The schematic diagram of the preparation of ethylcellulose microcapsules is shown in Figure 10.

### 4.6. The Morphology of Et(GB) and Et[Ch(GB)NP]

The microparticles’ morphology (the surface structure and shape) was evaluated using microphotography, obtained by means of a scanning electron microscope (SEM) at magnifications of 1000×, 1500×, 2000×, and 4000×. For the particles evaluated by SEM, the microcapsules were sprayed with gold and imaged on an FEI Quanta 200 model with the Q150R S vacuum sputtering machine (Hillsboro, OR, USA) to establish their morphological parameters.

### 4.7. GBE Release Kinetics from Nanoparticles—Ch(GB)NP and Microcapsules—Et(GB) and Et[Ch(GB)NP]

A Perkin Elmer UV-VIS spectrophotometer with Lambda 2 software (Waltham, MA, USA) was used to study the release kinetics of the core material (GBE). Samples of lyophilized Ch(GB)NP and dried ethylcellulose microcapsules were prepared in the form of tablets. A total of 0.05 g of each sample was weighed and then compressed into a tablet with a diameter of 12 mm and placed in 5 mL of the research medium (the studies were carried out in two research media, namely water and physiological saline). The studies were carried out at a temperature of 33 °C. Absorbance was measured at a wavelength characteristic for rutin, i.e., in the measurement range of 257–270 cm^−1^ (a calibration curve was previously determined). The measurements were taken at specific time intervals (5 min, 30 min, 1 h, 2 h, 4 h, 24 h, 96 h, 168 h, and 336 h) by taking 2 mL of the solution. The solution was refilled with fresh medium each time to the initial volume.

### 4.8. Statistical Analysis

Regression coefficient values (R^2^) were calculated for the release of GBE from both types of microcapsules into the research media (water and saline). Four different mathematical models (zero-order, first-order, Higuchi, and Korsmeyer–Peppas) were fitted with the obtained cumulative drug release and time curves to describe the kinetics. To determine which model was followed, the value of the regression coefficient (R^2^) was determined and compared.

## 5. Conclusions

Dual-layer natural polymer microcapsules containing plant extracts are a promising solution in several medical and industrial fields. By using polymeric layers, the bioactive substances contained in the extract are released in a controlled manner, increasing their stability and efficacy. Furthermore, the dual layers of natural polymers increase the resistance of the capsules to external factors such as pH, temperature, and enzymes, making them more versatile and durable carriers for a wide range of applications. In this paper, for the first time, the carriers were obtained using a matrix consisting of two polymers derived from natural sources, such as chitosan and ethyl cellulose, and a core material of *Ginkgo biloba* leaf extract (GBE). In addition, ethylcellulose microcapsules with chitosan nanoparticles and extract [Et[Ch(GB)NP] were characterized in terms of size, shape, surface morphology (SEM microscopy), and drug release kinetics (UV-VIS and mathematical release models). The nanotechnology-based structures exhibited broad-spectrum antimicrobial properties and slow-release kinetics. The encapsulation of GBE with a delayed-release polymeric double layer is a good alternative to preserve its components and potential antimicrobial properties so that it might be applied as a natural antimicrobial agent in the food industry.

## Figures and Tables

**Table 1:** Mathematical models and regression coefficient values of GBE release from Et[Ch(GB)NP].

Drug Release Model	Medium–Water (Sample A)	Medium–Saline (Sample B)
Zero-order	0.9450	0.9939
First-order	0.9472	0.9913
Higuchi	0.9738	0.9796
Korsmeyer–Peppas	0.7315	0.9538

**Table 2:** Mathematical models and regression coefficient values of GBE release from Et(GB).

Drug Release Model	Medium–Water (Sample A)	Medium–Saline (Sample B)
Zero order	0.7939	0.9945
First order	0.8012	0.9874
Higuchi	0.8865	0.9411
Korsmeyer–Peppas	0.9048	0.8169

**Table 3:** The list of tentatively identified components of the water–ethanol infusion from the *Ginkgo biloba* leaves, recorded in the negative ion mode (Rt—retention time, DBE—double bonds and ring number).

No	Ion	Rt [min]	Name of Compound	Neutral Molecular Formula	Theoretical Mass	Experimental Mass	MS/MS Fragments	Error of Measurement [ppm]	DBE
1	[M-H]-	1.9	Quinic acid	C_7_H_12_O_6_	191.0588	191.0579	173.0462 127.0410 111.0461	4.64	6.5
2	[M-H]-	3.005	Citric acid	C_6_H_8_O_7_	191.0197	191.0195	173.0108 129.0187 111.0079 87.0078	1.18	3
3	[M-H]-	7.841	Shikimic acid	C_7_H_10_O_5_	173.0455	173.0453	155.0357 130.9666 111.0460	1.42	3
4	[M-H]-	12.093	Protoca-techuic acid	C_7_H_6_O_4_	153.0193	153.0187	109.0288	4.1	5
5	[M-H]-	14.978	Coumaric acid glucoside	C_22_H_14_O_3_	325.0870	325.0857	163.0325 119.0434	4.04	16
6	[M-H]-	15.593	Acetylsy-ringic acid	C_11_H_12_O_6_	239.0561	239.0553	195.0649 179.0339 149.0598 133.0648	3.38	6
7	[M-H]-	17.014	Quercetin 3-O-α-L-rhamno-pyranosyl (1-2)[α-L-rhamno-pyranosyl(1-6)]-β-D-glucopyra-noside	C_33_H_40_O_20_	755.2040	755.2044	300.0262	−0.51	14
8	[M-H]-	17.347	Myricetin-3-O-rutinoside	C_27_H_30_O_17_	625.1410	625.1398	316.0207 271.0223	1.95	13
9	[M-H]-	17.597	Clitorin	C_33_H_40_O_19_	739.2091	739.2083	284.0312	1.08	14
10	[M-H]-	18.164	Rutoside	C_27_H_30_O_16_	609.1461	609.1467	564.4123 300.0282 271.0247	−0.97	13
11	[M-H]-	18.264/19.182/19.924/21.1	Ginkgolide isomers	C_20_H_24_O_10_	423.1297	423.1308	-	−2.66	9
12	[M-H]-	18.765	Kaempferol-3-O-β-D-rutinoside	C_27_H_30_O_15_	593.1512	593.1477	285.0348 255.0254	5.88	13
13	[M-H]-	19.349	Ginkgolide C or isomer	C_20_H_24_O_11_	439.1246	439.1221	383.1311 365.1197 321.1304 259.1307	5.65	9
14	[M-H]-	19.432	Ginkgolide Q or isomer	C_20_H_24_O_11_	439.1243	439.1209	411.1253 383.1311 321.1304 277.1403	8.37	9
15	[M-H]-	19.932	Bilobalide	C_15_H_18_O_8_	325.0929	325.0919	251.0869 237.1077 193.1179 163.1068	3.04	7
16	[M-H]-	21.5	Unknown	C_16_H_32_O_4_	287.2228	287.2222	287.2214	2.02	1
17	[M-H]-	21.633	Amento-flavone	C_30_H_18_O_10_	537.0827	537.0281	443.0403 413.0644 375.0495	1.15	22
18	[M-H]-	22.267	Bilobetin	C_31_H_20_O_10_	551.0984	551.0992	10 eV 387.0867 20 eV 519.0705 457.0552 413.0658 389.0665 323.0537	−1.5	22
19	[M-H]-	22.884	Ginkgetin	C_32_H_22_O_10_	565.1140	565.1136	533.0867 500.2757 389.0660 303.2317 151.0019	0.74	22
20	[M-H]-	23.435	Dirhamnosyl linolenic acid isomer	C_28_H_48_O_11_	559.3124	559.3113	513.3056 277.2157 253.0911	1.94	5
21	[M-H]-	24.102	Ginkgolic acid C17:2	C_24_H_36_O_3_	371.2439	371.2456	-	−4.53	3
22	[M-H]-	24.219	Sciado-pitysin	C_33_H_24_O_10_	579.1297	579.1324	547.1023 232.0829 503.0738 403.0802 165.0167	−4.7	22
23	[M-H]-	24.269	Ginkgolic acid C13:0	C_20_H_32_O_3_	319.2279	319.2287	-	−2.6	5
24	[M-H]-	26.604	Unknown	C_22_H_34_O_4_	361.2384	361.2370	317.2490 299.2383 287.2385 245.1906 231.1759	3.96	6

## Data Availability

The data presented in this study are available upon request from the corresponding author.

## Supplementary Materials

The following supporting information can be downloaded at https://www.mdpi.com/article/10.3390/ijms26073066/s1.
