# Supplementary material for: Dual Biopolymer Layer Using Nanoparticles with Active Substance Enclosed in Microcapsules: Innovative Solution for Slow Release of Ginkgo biloba L. Extract for Potential Therapies
Source: Int J Mol Sci. 2025 Mar 27;26(7):3066. doi: 10.3390/ijms26073066 (PMC11988488; doi:10.3390/ijms26073066)
Supplement: Supplementary file 1 [file ijms-26-03066-s001.zip › ijms-3513669-supplementary.pdf]

Supplementary File

Table S1. The MS/MS spectra of the tentatively identified compounds in the gingko tincture obtained in the HPLC-ESI-QTOF-MS/MS analysis.

<table>
<tr><th>No</th><th>Name of compound</th><th>MS/MS fragments</th></tr>
<tr><td>1</td><td>Quinic acid</td><td>
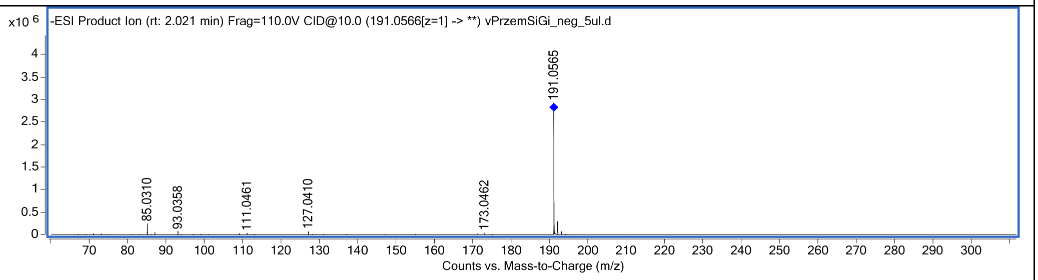

</td></tr>
<tr><td>2</td><td>Citric acid</td><td>
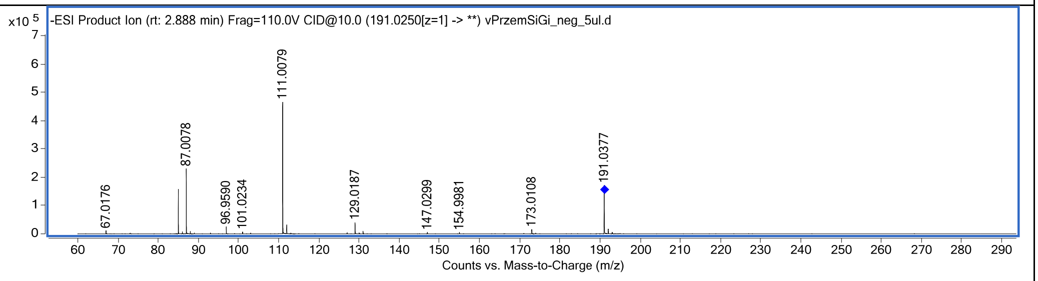

</td></tr>
<tr><td>3</td><td>Shikimic acid</td><td>
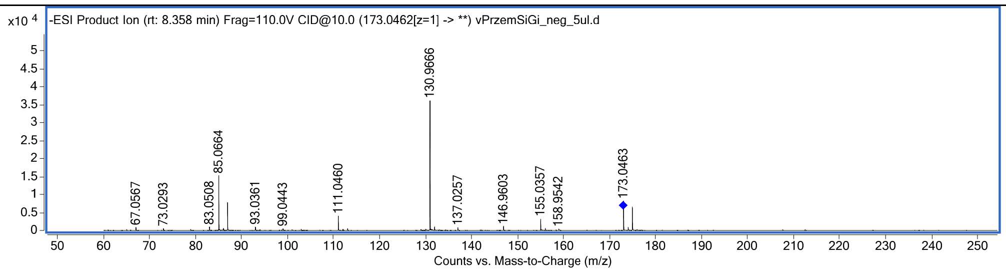

</td></tr>
</table>

<table>
<tr><td>4</td><td>Protocatechuic acid</td><td>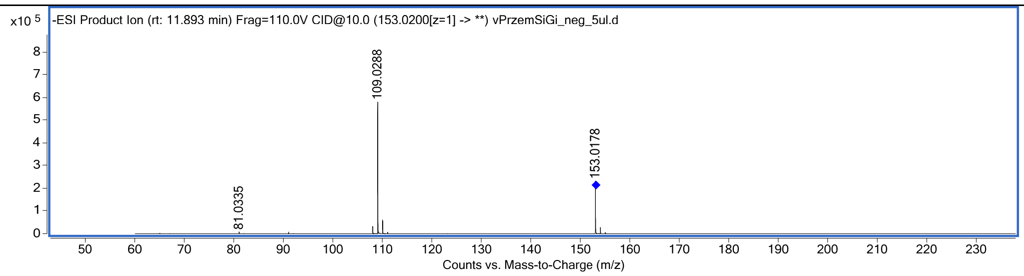
</td></tr>
<tr><td>5</td><td>Coumaric acid glucoside</td><td>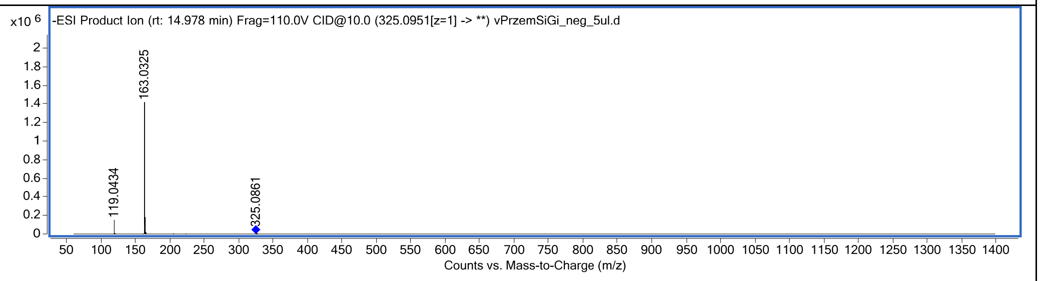
</td></tr>
<tr><td>6</td><td>Acetylsyringic acid</td><td>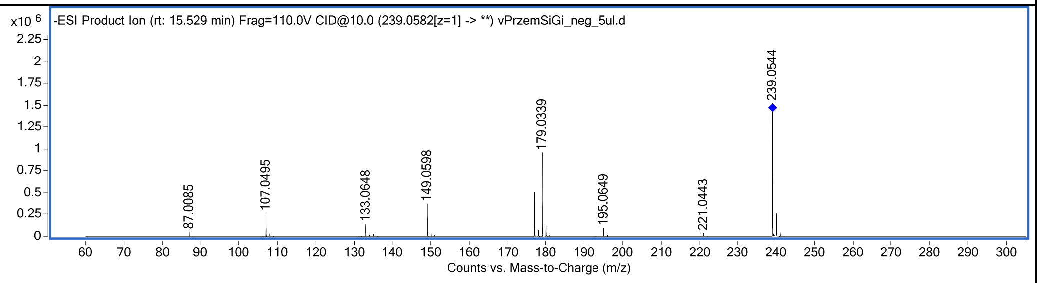
</td></tr>
<tr><td>7</td><td>Quercetin 3-O-α-L-rhamnopyranosyl(1-2)[α-L-rhamnopyranosyl(1-6)]-β-D-glucopyranoside</td><td>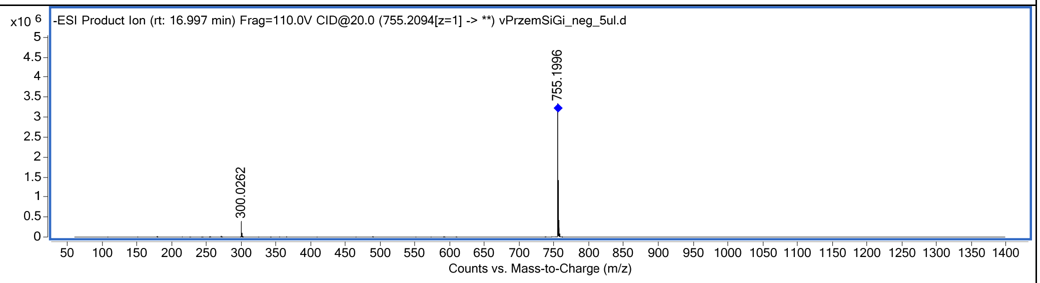
</td></tr>
</table>

8	Myricetin-3-O-rutinoside	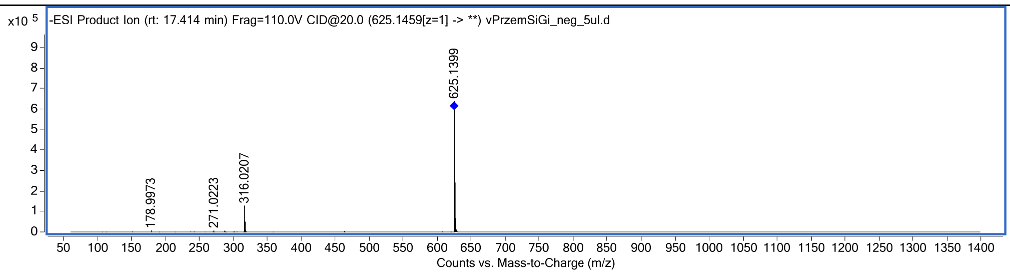
9	Clitorin	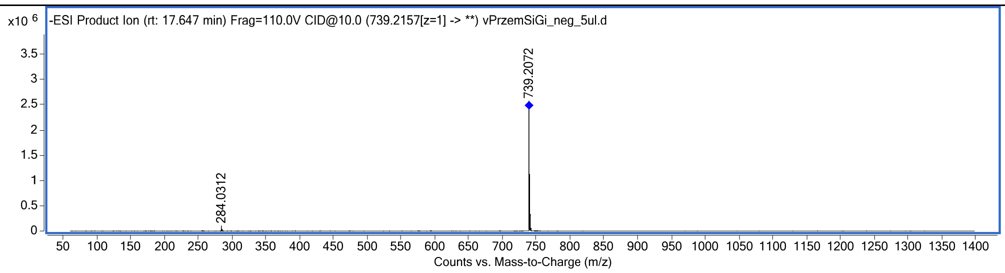
10	Rutoside	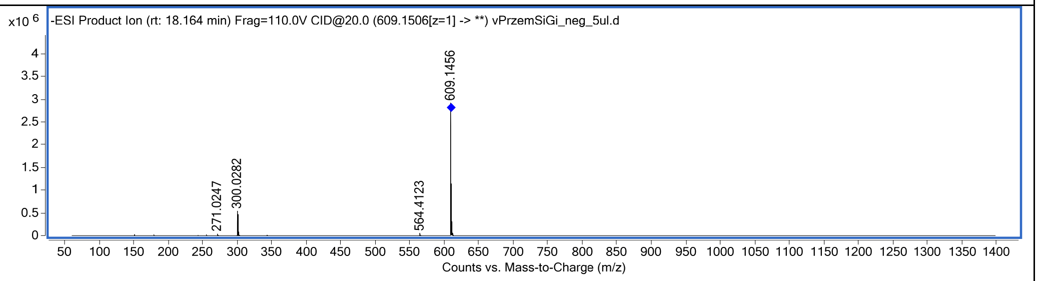
11	Ginkgolide isomers	-
12	Kaempferol-3-O-β-D-rutinoside	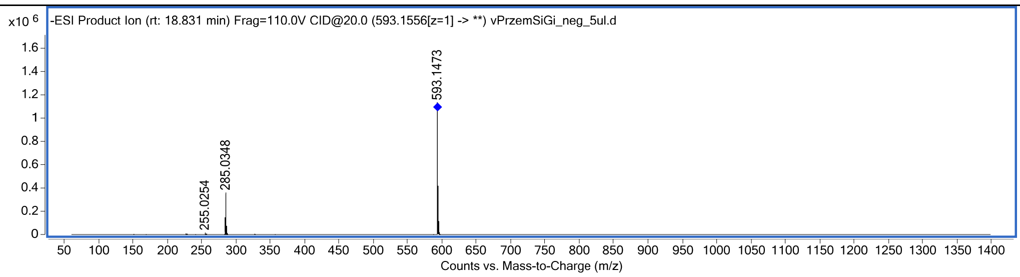

13	Ginkgolide C or isomer	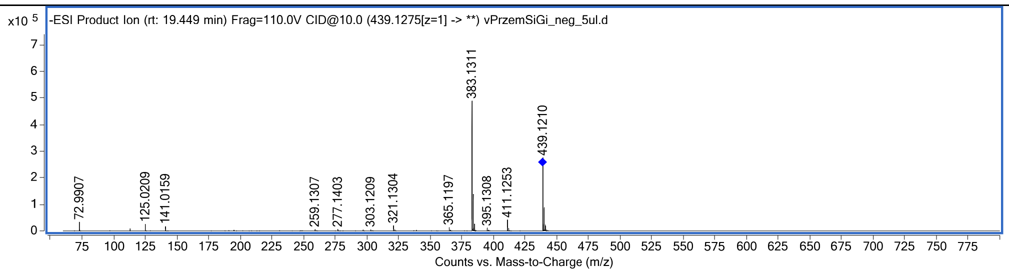
14	Ginkgolide Q or isomer	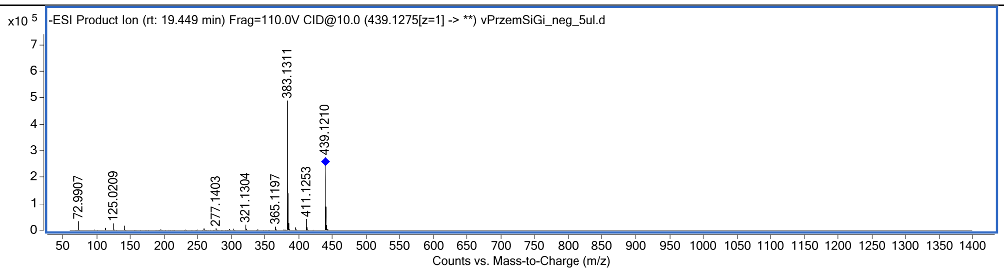
15	Bilobalide	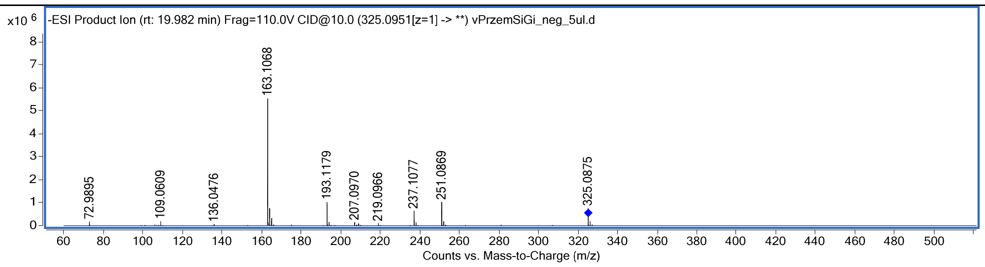
16	Unknown	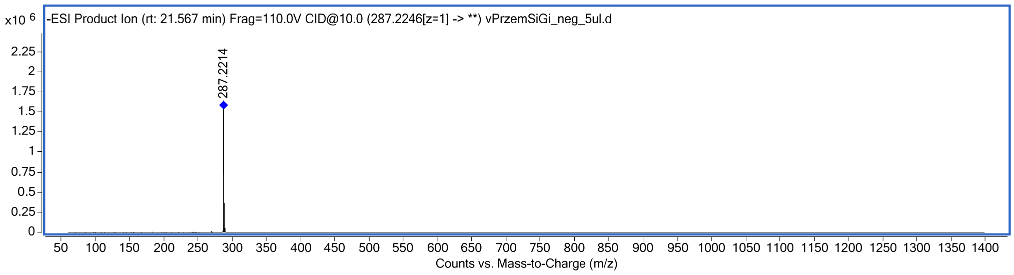

<table>
<tr><td>17</td><td>Amentoflavone</td><td>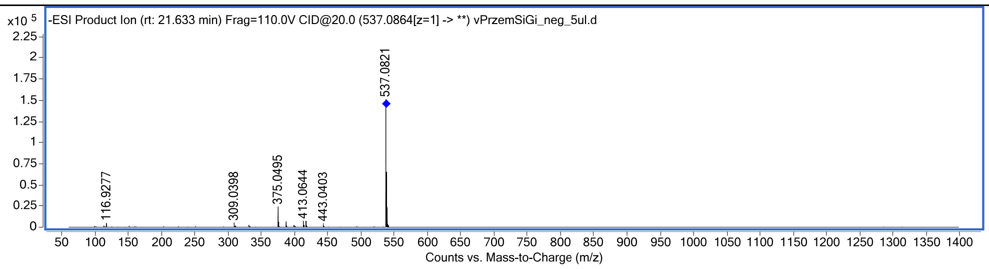
</td></tr>
<tr><td>18</td><td>Bilobetin</td><td>10 eV
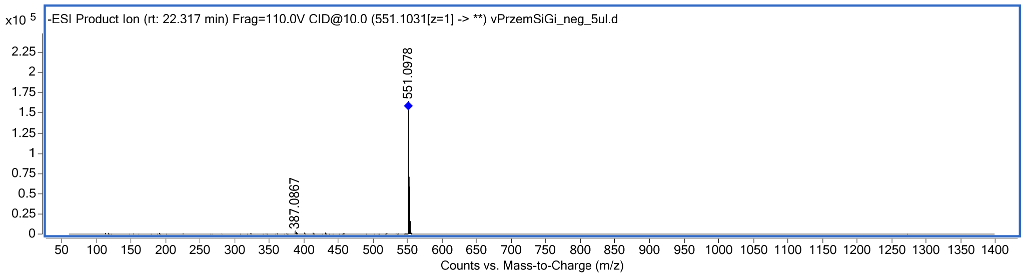

20 eV
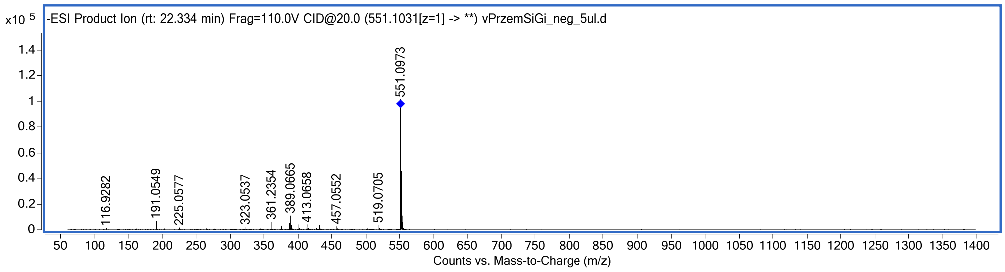
</td></tr>
<tr><td>19</td><td>Ginkgetin</td><td>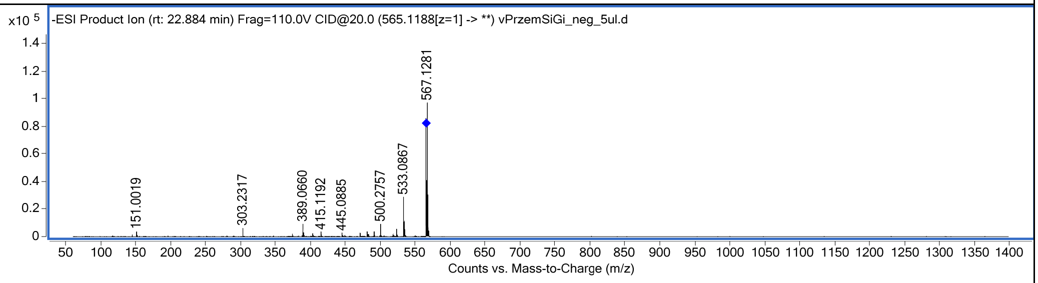
</td></tr>
</table>

20	Dirhamnosyl linolenic acid isomer	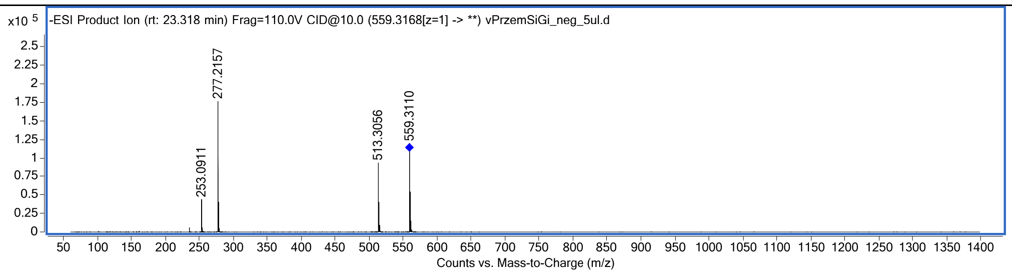
21	Ginkgolic acid C17:2	-
22	Sciadopitysin	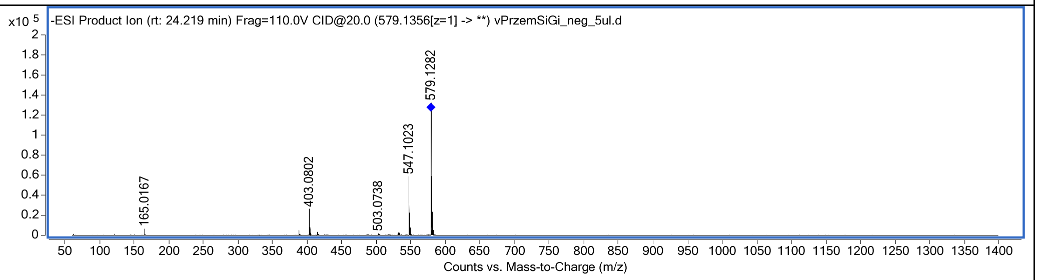
23	Ginkgolic acid C13:0	-
24	Unknown	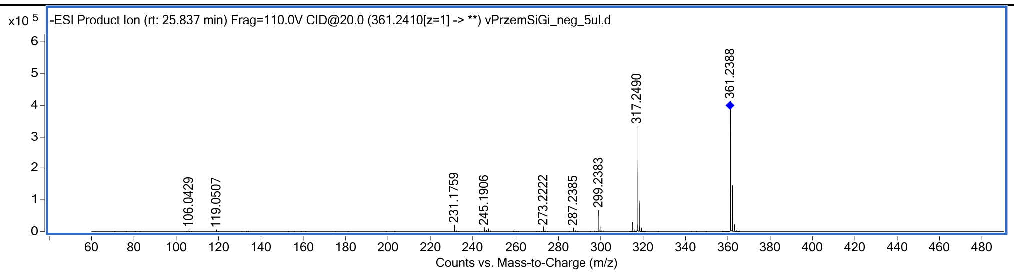
